# The effect of zinc supplementation on glucose homeostasis: a randomised double-blind placebo-controlled trial

**DOI:** 10.1007/s00592-022-01888-x

**Published:** 2022-04-22

**Authors:** John R. Attia, Elizabeth Holliday, Natasha Weaver, Roseanne Peel, Kerry C. Fleming, Alexis Hure, John Wiggers, Mark McEvoy, Andrew Searles, Penny Reeves, Priyanga Ranasinghe, Ranil Jayawardena, Samir Samman, Judy Luu, Chris Rissel, Shamasunder Acharya

**Affiliations:** 1 School of Medicine and Public Health, University of Newcastle, New Lambton Heights, Australia; 2 Division of Medicine, Hunter New England Local Health District, New Lambton Heights, Australia; 3 School of Medicine and Public Health, University of Newcastle, New Lambton Heights, Australia; 4 Diabetes Service and Diabetes Alliance Hunter New England Local Health District, New Lambton Heights, Australia; 5 Hunter New England Local Health District, New Lambton Heights, Australia; 6 La Trobe Rural Health School, College of Science, Health and Engineering, La Trobe University, Bendigo, VIC Australia; 7 Hunter Medical Research Institute, New Lambton Heights, Australia; 8 Faculty of Medicine, University of Colombo, Colombo, Sri Lanka; 9 School of Life and Environmental Sciences, University of Sydney, New Lambton Heights, Australia; 10 Diabetes Stream, Diabetes Alliance HNELHD, New Lambton Heights, Australia; 11 The University of Sydney, Camperdown, 2050 Australia; 12 Endocrinology and Diabetes Service and Diabetes Alliance Hunter New England Local Health District, New Lambton Heights, Australia

**Keywords:** Prediabetes, Zinc supplementation, Randomized controlled trial, Prevention, Diabetes prevention, General practice, Get healthy, Adults, Australia

## Abstract

**Aims:**

The burden and health costs of Type 2 Diabetes Mellitus continue to increase globally and prevention strategies in at-risk people need to be explored. Previous work, in both animal models and humans, supports the role of zinc in improving glucose homeostasis. We, therefore, aimed to test the effectiveness of zinc supplementation on glycaemic control in pre-diabetic adults.

**Methods:**

We conducted a randomized, double-blind, placebo-controlled trial across 10 General Practitioner (GP) practices in NSW, Australia. The trial is known as Zinc in Preventing the Progression of pre-Diabetes (ZIPPeD)Study. Pre-diabetic (haemoglobin A1c [HbA1c] 5.7–6.4%, 39–46 mmol/mol) men and women (*N* = 98) were all assigned to a free state government telephone health coaching service (New South Wales Get Healthy Information and Coaching Service) and then randomised to either daily 30 mg zinc gluconate or placebo. Blood tests were collected at baseline, 1, 6 and 12 months for the primary outcomes (HbA1c, fasting blood glucose (FBG)); secondary outcomes included Homeostasis Model Assessment 2 (HOMA 2) parameters, lipids, body weight, height, waist circumference, blood pressure and pulse.

**Results:**

The baseline-adjusted mean group difference at 6 months, expressed as treatment–placebo, (95% CI) was −0.02 (−0.14, 0.11, *p* = 0.78) for HbA1c and 0.17 (−0.07, 0.42; *p* = 0.17) for FBG, neither of which were statistically significant. There were also no significant differences between groups in any of the secondary outcomes. Zinc was well tolerated, and compliance was high (88%).

**Conclusion:**

We believe our results are consistent with other Western clinical trial studies and do not support the use of supplemental zinc in populations with a Western diet. There may still be a role for supplemental zinc in the developing world where diets may be zinc deficient.

**Trial registration:**

Australian and New Zealand Clinical Trials Registry, ACTRN12618001120268. Registered on 6 July 2018.

**Supplementary Information:**

The online version contains supplementary material available at 10.1007/s00592-022-01888-x.

## Background and aims

As of 2019, 9.3% of the world population (463 million people aged 20–79 years) had a diagnosis of diabetes; this is projected to increase to 578 million by 2030 and 700 million by 2045 [1]. The global cost to health systems in 2019 was $760 billion and is projected to increase to over $825 billion (USD) by 2030, and $845 billion by 2045 [1]. It is estimated that 90–95% of all cases of diabetes are type 2 [2] and, given that the natural history of this chronic condition is to first pass through a stage of impaired glucose tolerance (pre-diabetes), there is an opportunity to intervene early, thereby delaying progression to diabetes, and avoiding later consequences including retinal disease, kidney disease, peripheral vascular disease, and cardiovascular disease [3, 4].

Type 2 Diabetes is largely preventable by improving lifestyle factors including nutrition, physical activity and maintenance of normal body weight; these measures should obviously be targeted at the entire population, not only for the prevention of diabetes but also other chronic diseases [5]. However, programs that target these behaviours takes time to show an effect and sustained health behaviour change is slow. Therefore, cheap and simple interventions that can prevent or at least delay the progression of disease while these health behaviour interventions are adopted would be a useful adjunct in our preventive measures.

We previously outlined the various roles of zinc in glucose handling [6], including being involved in the synthesis, storage, and secretion of insulin, and moderating inflammatory cytokines [7–10]. Oxidative stress is relatively common in diabetes, and zinc also has anti-oxidant properties, acting as a cofactor for the superoxide dismutase enzyme that detoxifies reactive oxygen species [11]. Genome-wide association studies of Type 2 Diabetes have also identified an association with a genetic variant in the *SLC30A8* gene, which encodes a zinc transporter, ZnT8 [12]. The associated genetic variant causes an amino acid change in the transporter which increases disease risk by an estimated 17% per risk allele [13].

Zinc supplementation studies in animal models of diabetes over the last 35 years have been largely positive and support the use of supplemental zinc to improve glucose handling [6, 14–17]. Meta-analyses of zinc supplementation in humans have also suggested favourable effects on fasting blood glucose (FBG) and Haemoglobin A1c (HbA1c) [18, 19]. The majority of the trials identified by these two reviews, however, are small (20–30 participants per group), short-term (up to 6–12 weeks of supplementation), and in low- and middle-income countries (Iran, Iraq, India, Sri Lanka) where diets may be deficient in zinc.

Perhaps the most compelling evidence for the role of supplemental zinc in preventing the progression of pre-diabetes is the RCT conducted by Ranasinghe et al. [20] in Sri Lanka (SLCTR/2012/010). Over 12 months, 20 mg of daily zinc supplementation significantly improved FBG, Oral Glucose Tolerance Test (OGTT), and Homeostasis Model Assessment (HOMA)-calculated insulin resistance and beta-cell function compared to the placebo group. Furthermore, the progression to overt diabetes was reduced from 25% in the placebo group to 11% in the zinc group (*p* = 0.016) [6].

We set out to test whether zinc supplementation was effective in a pre-diabetic population with a Western diet, trialing 30 mg/day supplemental zinc for 12 months, with primary endpoints of HbA1c and FBG at 6 months.

## Methods

We conducted a randomised, double-blind, placebo-controlled trial in a pre-diabetic population aged 40–70 years; zinc supplementation (30 mg/daily) or placebo was continued over 12 months, with blood samples taken at baseline, 1, 6 and 12 months for FBG, HbA1c, and insulin levels for calculation of HOMA parameters. The two primary outcomes were HbA1c and FBG, each measured after 6 months of treatment. The trial is known as Zinc in Preventing the Progression of pre-Diabetes (ZIPPeD Study). The protocol has been previously published (Trial registration: ACTRN12618001120268) with methods briefly described below [6].

### Recruitment

The study was conducted in Newcastle (NSW, Australia) through the Hunter Diabetes Alliance, which brings together hospital-based diabetes specialists and general practitioners in the Hunter New England local health district to improve diabetes care, using a case conferencing model of continuing professional development [6, 21]. Practices that were part of the Alliance were invited to participate in this study. A research nurse worked with each practice nurse to identify pre-diabetic patients using a two-fold approach:

- Review of practice records over the previous year for HbA1c or FBG values in the pre-diabetic range (5.7–6.4% (39–46 mmols/mol) and 6.1–6.9 mmol/l respectively)
- Screening of patients in the waiting room using the AUSDIAB questionnaire (AUSDRISK) [22] and a point of care HbA1c.

### Eligibility criteria

- Age = 40–70 years
- Pre-diabetes as defined by HbA1c of 5.7–6.4%, (39–46 mmols/mol)
- Body Mass Index (BMI) ≥ 27 kg/m^2^

### Exclusion criteria

- Taking any other vitamin or mineral supplementation containing zinc
- Currently using weight loss medication
- History of Diabetes Mellitus.
- Pregnancy or lactation for women of child-bearing age
- Impaired hepatic (AST or ALT > three times the upper limit of normal) or renal function (Stage 3 chronic kidney disease)
- Taking pharmacological agents that may interfere with the intervention (for example, diuretics, metformin, and complementary medicines)
- Past history of pancreatitis
- Current cancer under treatment, terminal cancer, terminal illness

### Baseline visit and randomization

Patients meeting the above criteria were invited to participate. Those giving informed consent had their height, weight, and hip circumference measured in the practice and were provided with pathology forms to check their baseline FBG, HbA1c, and insulin levels, as well as lipid profile at their local public pathology provider, with whom the team had a research agreement. A baseline questionnaire captured socio-demographic information, smoking and alcohol consumption, ethnicity, self-reported medical history, medications, and exercise. Diet quality was captured using a web-based food frequency questionnaire (FFQ) [23]. Every participant was also referred to the Get Healthy Information and Coaching Service, run by the New South Wales state health department. This free Service provides up to 13 telephone calls over 6 months from allied health professionals who provide personalised health information and support participants to achieve a better lifestyle focusing on diet, physical activity, smoking cessation and alcohol reduction [24].

Randomisation codes were generated by an independent statistical team (The Clinical Research Design, Information Technology and Statistical Support (CReDITSS) unit at the Hunter Medical Research Institute) using permuted blocks of size 4 or 6, stratified by General Practitioner (GP) practice. These were entered into a Research Electronic Data Capture (REDCap) database [25] and assigned sequentially by the research manager (independent of the study nurse).

### Intervention

On completion of all the baseline measures, participants were randomised to the treatment or placebo group. Active drug was a daily capsule containing 30 mg elemental zinc gluconate, based on the dose from previous studies [2], our pilot data, and the mean dose in the meta-analysis [18] (donated by Blackmores Ltd, Australia). The placebo capsule, containing cellulose, was identical in appearance, shape, and colour to the zinc capsule (also donated by Blackmores Ltd. Australia). All study participants were sent a 3-month supply, with instructions to take the study capsule daily with breakfast. Every quarter thereafter, participants were sent a new 3-month supply with instructions to return a side-effect questionnaire and any remaining capsules via a postage-paid envelope; adherence was assessed by the study nurse using pill counts of the returned bottles and by calculating the percentage of total tablets taken during the measured interval.

### Follow-up

Participants were followed up every 3 months by mail with a new supply of capsules and an adverse event form to complete. A reply-paid padded envelope was included for the return of old pill bottles and the completed adverse event report. Pathology request forms were mailed out at 1-, 6- and 12-month timepoints. Reminders for baseline and follow-up blood tests and other incomplete tasks (surveys) were sent by SMS, postcard, phone call or email as required. At the final 12-month timepoint, blood was collected and analysed for lipid profile. Height, weight and waist circumference were measured again at 12 months at the general practice, and the web based FFQ was re-administered.

### Outcome measures

#### Primary outcome measures

The two primary outcomes were HbA1c and FBG at 12 months.

#### Secondary measures

The secondary outcomes were:

- HbA1c and FBG at 1 month and 6 months
- HOMA parameters of beta cell function, insulin resistance, and insulin sensitivity at 1 month, 6 months and 12 months (calculated using the Homeostasis Model Assessment 2(HOMA2)) calculator University of Oxford:
  http://www.dtu.ox.ac.uk/homacalculator/index.php).
- Lipid profile at 1 month, 6 months and 12 months
- Weight, BMI, waist circumference and blood pressure at 12 months
- Progression to diabetes at 12 months (as defined by HbA1c >  = 6.5%, (> 48mmols/mol) or fasting blood glucose >  = 7 mmol/l)
- Adherence (defined as % of total required capsules taken)

### Measurement of biochemical variables

Blood samples were collected from participants and analysed as contracted by Pathology North, a NATA-accredited public pathology provider associated with outlets across the study catchment.

### Sample size

The sample size was based on the primary or secondary endpoint with the smallest assumed effect size: insulin sensitivity (IS). We estimated that with an assumed mean difference in IS of 0.3 (with SD of 0.7, equal to Cohen’s d of 0.4) between the intervention and control groups, we would need 164 participants per group to reject the null hypothesis with probability (power) 0.9 and type I error probability (α) of 0.01. Allowing up to 20% loss to follow-up over 1 year, we aimed to recruit a total of 410 participants. Unfortunately, COVID-19 restrictions curtailed our ability to visit GP practices and recruit face to face. The study was ended prematurely with 98 participants randomised. These participants were all followed up for 1 year.

### Statistical analyses

Distributions of participant baseline characteristics were summarised by group using mean with standard deviation (SD), median with interquartile range (Q1, Q3) or frequency with percent, as appropriate.

For primary outcomes, distributions were summarised using mean (SD) for each drug group at baseline, 1 month, 6 months and 12 months. Treatment effects were estimated using a linear mixed model including outcomes measured at all three follow-up timepoints. Models included a random intercept for individual (to account for repeated measures on participants) and fixed effects for group, time (categorical), and the interaction term of group x time. Models were adjusted for sex and the baseline value of the outcome. Group differences were reported as the baseline-adjusted mean group difference at 1 month, 6 months and 12 months with 95% confidence interval (CI) and *p*-value. A type III *p*-value for the interaction term was also presented. For each of the two primary endpoints (6 months), two-sided statistical significance was evaluated at the 0.025 level. A scatter plot of fitted values versus studentized residuals and a needle plot of Cook’s distances was used to check for influential outliers.

In a secondary analysis of the primary outcomes, a spaghetti plot (participant outcome values versus continuous time) was used to assess linearity of the time effect, which was judged as fair. Models were then refit including time as a continuous predictor, assuming linearity, and the group effect was estimated as a difference in slopes (Drug B vs. Drug A) with 95% CI.

For secondary outcomes measured at baseline, 1 month, 6 months and 12 months, analyses were performed as for the primary outcomes, with statistical significance declared at the 0.05 level. For secondary outcomes measured at baseline and 12 months only (body weight, BMI, waist circumference, blood pressure), baseline-adjusted group differences at 12 months were estimated using a linear model, adjusted for baseline values, with no random effect or interaction term.

Analyses were performed in SAS 9.4 (SAS Institute, Cary, NC, USA) using the MIXED procedure with REML estimation algorithm. LSMEANS statements were used to estimate the mean differences and their CIs.

## Results

**Fig. 1:**
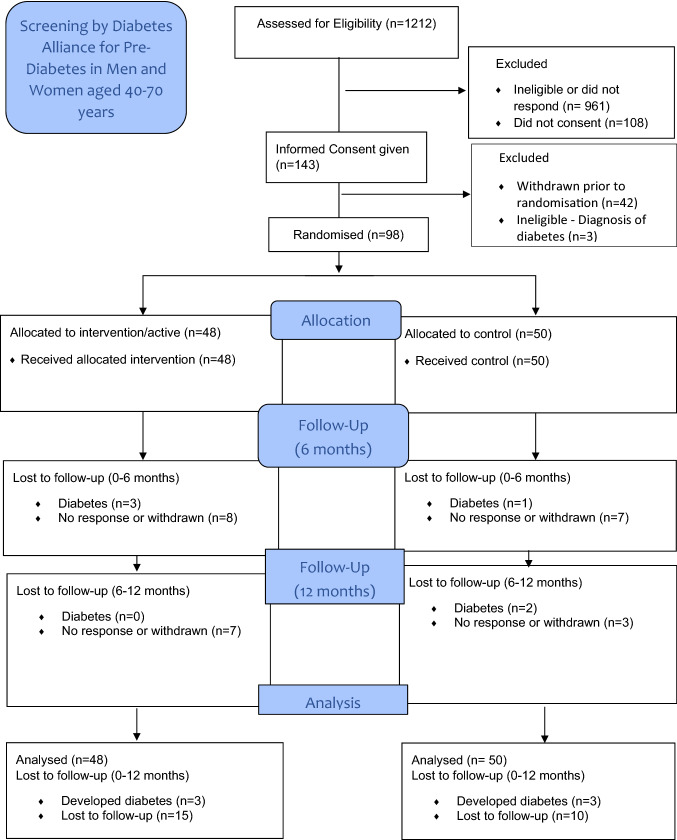
Zinc In Preventing the Progression of pre-Diabetes Study (ZIPPeD Study) Consort Flow Diagram

We identified 1212 people with pre-diabetes across 10 practices, of which 961 were non-eligible or non-contactable; 251 were approached, of which 143 consented but only 98 completed baseline questionnaires and were subsequently randomised. Of the remaining 45, 3 were found to be diabetic and hence ineligible and 42 never completed baseline questionnaires or bloods and so did not progress to randomisation; the consort flow diagram summarising recruitment is shown in Fig. 1.

These participants consisted of 44 males and 54 females (*n* = 98, 55% female); age was slightly left-skewed with mean (SD) 60.8 (7.5) and median (IQR) 62.9 (56.6, 67.0). Mean (SD) BMI was 34.1 (6.3) and mean waist circumference was largely above the healthy recommended limit of 88 cm in women and 102 in men. Around half of participants had never smoked (*n* = 52, 53%), and around half consumed alcohol within recommended levels.

The study participant characteristics were well balanced across the 2 arms of the study (see Table 1).

**Table 1:** Characteristics of randomised patients

Characteristic	Class or Statistic	Treatment group	
		Placebo (*n* = 50)	Active (*n* = 48)
Age	median (Q1, Q3)	63.6 (58.9, 67.2)	61.6 (54.4, 66.2)
Sex	Female	27 (54%)	27 (56%)
Highest level of education	Primary schooling only		1 (2.1%)
	Secondary schooling not completed	9 (18%)	12 (25%)
	Secondary schooling completed	17 (34%)	9 (19%)
	Trade qualification or TAFE	15 (30%)	19 (40%)
	University or other tertiary study	9 (18%)	7 (15%)
Smoking	Never smoked	27 (54%)	25 (52%)
	Current smoker	5 (10%)	8 (17%)
	Past smoker	18 (36%)	15 (31%)
Alcohol use meets NHMRC guideline	Yes	26 (52%)	26 (54%)
Taking any medication	Yes	43 (88%)	36 (75%)
	Aspirin/antiplatelet	7 (14%)	7 (15%)
	Blood pressure	24 (49%)	22 (46%)
	Cholesterol	21 (43%)	16 (33%)
	Anticoagulant	2 (4.1%)	2 (4.2%)
Systolic blood pressure	Mean (SD)	134.5 (16.3)	133.1 (17.0)
Diastolic blood pressure	Mean (SD)	82.3 (9.2)	83.4 (9.4)
Height	Mean (SD)	167.3 (8.8)	166.7 (10.3)
Body weight	Mean (SD)	94.5 (20.1)	95.6 (17.0)
BMI (Body Mass Index)	Median (Q1, Q3)	31.6 (29.9, 38.6)	33.8 (30.7, 37.6)
Waist Circumference	Mean (SD)	110.1 (14.9)	109.8 (12.0)
Walking (mins per week)	Median (Q1, Q3)	120.0 (40.2, 225.0)	120.0 (33.0, 232.5)
Moderate physical activity (mins per week, equivalent)	Median (Q1, Q3)	160.5 (18.0, 360.0)	210.0 (30.0, 480.0)
HDL	Median (Q1, Q3)	1.3 (1.0, 1.5)	1.2 (1.1, 1.5)
LDL	Mean (SD)	3.1 (1.0)	3.0 (0.9)
Total cholesterol	Mean (SD)	5.1 (1.0)	5.1 (1.1)
Triglycerides	Median (Q1, Q3)	1.5 (1.3, 2.1)	1.5 (1.2, 2.2)
Fasting blood glucose level	Mean (SD)	5.9 (0.8)	5.8 (0.7)
HbA1c %	Median (Q1, Q3)	5.9 (5.7, 6.0)	5.8 (5.7, 5.9)

The baseline-adjusted mean group difference, expressed as treatment–placebo (95% CI) for HbA1c at 6 months was −0.02 (−0.14, 0.11) and was not statistically significant (*p* = 0.79). The baseline-adjusted mean group difference in FBG at 6 months was also not statistically significant, at 0.17 (−0.07, 0.42; *p* = 0.17). The type III p-value indicated no global difference in HbA1c across the three follow-up timepoints (*p* = 0.42). The type III *p*-value for FBG was 0.034, but group differences did not show a consistent direction of effect across timepoints (see Table 2).

**Table 2:** Analysis of primary outcomes

Outcomes	Time	Treatment group		Active-placebo comparison		
		Placebo	Active	Mean difference (95% CI)	*P*-value	Type III *P*-value
*Primary outcomes*						
HbA1c	Baseline	5.90 (0.20)	5.87 (0.19)			
	1 month	5.85 (0.35)	5.82 (0.25)	− 0.00 (− 0.12, 0.12)	0.9969	
	6 month	5.86 (0.24)	5.82 (0.29)	− 0.02 (− 0.14, 0.11)	0.7871	
	**12 month**	**5.96 (0.30)**	**5.84 (0.29)**	** − 0.06 (− 0.19, 0.06)**	**0.3214**	**0.4167**
Fasting blood glucose level	Baseline	5.88 (0.84)	5.81 (0.71)			
	1 month	5.67 (0.63)	5.66 (0.68)	0.04 (− 0.19, 0.26)	0.7540	
	6 month	5.61 (0.65)	5.75 (0.71)	0.17 (− 0.07, 0.42)	0.1691	
	**12 month**	**5.89 (0.66)**	**5.59 (0.68)**	** − 0.19 (− 0.44, 0.06)**	**0.1386**	**0.0336**

Bold values indicate the main outcome timeframe

A small number of the group differences in secondary outcomes were statistically significantly at the 0.05 level, either evaluated at single timepoints, or across all follow-up timepoints (type III *p*-value) but these were not in any consistent directions and are likely due to type I error. Table 3 shows effect estimates for HOMA-derived parameters of beta-cell function, insulin resistance, insulin sensitivity and lipid parameters.

**Table 3:** Analysis of secondary outcomes (HOMA2 parameters and laboratory measurements)

Outcomes	Time	Treatment group		Active-placebo comparison		
		Placebo	Active	Mean difference (95% CI)	*P*-value	Type III *P*-value
Beta cell function (%B)	Baseline	105.01 (36.81)	100.37 (35.38)			
	1 month	113.08 (39.86)	107.48 (34.73)	− 5.74 (− 16.71, 5.23)	0.3028	
	6 month	114.47 (44.71)	102.43 (30.09)	− 10.01 (− 21.64, 1.63)	0.0913	
	12 month	105.83 (36.60)	101.40 (29.04)	− 2.71 (− 14.56, 9.14)	0.6521	0.4306
Insulin sensitivity (%S)	Baseline	69.82 (44.30)	76.22 (44.39)			
	1 month	67.43 (37.03)	74.05 (44.35)	6.96 (− 3.51, 17.43)	0.1910	
	6 month	74.07 (52.00)	70.89 (34.51)	− 1.30 (− 12.53, 9.93)	0.8195	
	12 month	65.71 (34.84)	77.70 (34.58)	12.17 (0.68, 23.65)	0.0381	0.0930
Insulin resistance (IR)	Baseline	1.89 (0.91)	1.74 (0.94)			
	1 month	1.91 (1.04)	1.76 (0.83)	− 0.14 (− 0.37, 0.10)	0.2597	
	6 month	1.90 (1.08)	1.73 (0.79)	− 0.11 (− 0.36, 0.15)	0.4013	
	12 month	1.96 (1.02)	1.58 (0.74)	− 0.26 (− 0.52, − 0.00)	0.0463	0.4923
Total cholesterol (mmol/l)	Baseline	5.13 (1.04)	5.14 (1.09)			
	1 month	5.06 (1.00)	5.10 (1.23)	0.11 (− 0.19, 0.41)	0.4764	
	6 month	4.98 (1.00)	5.35 (1.37)	0.26 (− 0.05, 0.58)	0.1042	
	12 month	4.87 (1.08)	5.12 (1.41)	0.10 (− 0.22, 0.43)	0.5367	0.5667
LDL (mmol/l)	Baseline	3.08 (0.98)	3.01 (0.88)			
	1 month	2.97 (0.90)	3.09 (1.03)	0.14 (− 0.12, 0.41)	0.2861	
	6 month	3.02 (0.97)	3.25 (1.16)	0.12 (− 0.16, 0.40)	0.3894	
	12 month	2.90 (0.97)	3.04 (1.20)	0.06 (− 0.23, 0.35)	0.6797	0.8359
HDL (mmol/l)	Baseline	1.28 (0.34)	1.29 (0.36)			
	1 month	1.28 (0.32)	1.25 (0.33)	− 0.03 (− 0.16, 0.11)	0.6917	
	6 month	1.42 (0.70)	1.30 (0.36)	− 0.15 (− 0.30, − 0.00)	0.0446	
	12 month	1.26 (0.30)	1.32 (0.35)	0.01 (− 0.14, 0.16)	0.9158	0.2546
Triglycerides (mmol/l)	Baseline	1.74 (0.77)	1.86 (1.07)			
	1 month	1.70 (0.72)	1.69 (0.72)	− 0.05 (− 0.31, 0.20)	0.6851	
	6 month	1.55 (0.78)	1.90 (0.86)	0.29 (0.02, 0.56)	0.0358	
	12 month	1.67 (0.83)	1.70 (0.81)	0.04 (− 0.23, 0.31)	0.7774	0.0158

Table 4 shows estimates for outcomes of metabolic health, including weight, BMI, waist circumference and blood pressure.

**Table 4:** Analysis of secondary outcomes (physical measurements)

	Treatment group		Active—placebo		
Outcomes	Timepoint	Placebo	Active	Mean difference (95% CI)	*P*-value (treatment)
Body weight kilograms	Baseline	94.53 (20.07)	95.60 (17.00)		
	12 month	93.86 (20.73)	92.28 (16.16)	− 0.60 (− 2.51, 1.31)	0.5330
BMI (Body Mass Index) kg/m^2^	Baseline	33.58 (5.72)	34.57 (6.80)		
	12 month	32.85 (6.75)	32.74 (5.17)	− 0.07 (− 0.87, 0.73)	0.8661
Waist Circumference (cm)	Baseline	110.13 (14.95)	109.80 (11.96)		
	12 month	108.57 (16.58)	106.53 (10.60)	− 0.99 (− 4.32, 2.33)	0.5528
Systolic blood pressure (mmHg)	Baseline	134.52 (16.28)	133.10 (16.97)		
	12 month	133.23 (14.90)	132.42 (13.57)	− 1.89 (− 8.58, 4.80)	0.5741
Diastolic blood pressure (mmHg)	Baseline	82.26 (9.16)	83.38 (9.35)		
	12 month	78.09 (8.87)	79.55 (8.19)	− 0.39 (− 4.18, 3.40)	0.8369

Zinc was generally well tolerated and adherence was high; percentage of all doses taken was 88% (± 10%) for the zinc group vs 86% (± 12%) for the placebo group; this translates into 66% of the placebo group having at least 80% compliance versus 63% in the active group. Not all participants completed the side effect questionnaire, but for those who did dry mouth, heartburn, indigestion, stomach pain, diarrhoea, cramping were the most common symptoms and were equally distributed across both groups. There were no severe adverse events. (See Supplemental Table S1).

A sensitivity analysis assessing whether greater adherence with the zinc capsules was associated with better FBG or HbA1c using linear regression did not find any significant effect. Each 1% increase in adherence was associated with an estimated 0.55% (95% CI −0.63–1.73, *p* = 0.35) increase in HbA1c and a 0.94 mmol/mol (95% CI −1.36–3.24, *p* = 0.41) increase in FBG.

## Discussion

We did not find any evidence that zinc supplementation in an Australian cohort of pre-diabetic individuals over the course of 12 months had any effect on glucose handling, whether measured by FBG, HbA1c or HOMA parameters; there was also no difference on any metabolic measures, whether measured by lipid profile, blood pressure, BMI, or waist circumference. How can we reconcile these results with the body of literature demonstrating an effect of zinc?

Although recruitment was ceased prematurely due to COVID-19, our study still represents one of the largest RCTs in this area with the longest follow-up, and we do not believe that type II error, i.e. lack of power, is a likely explanation for our negative result. The meta-analyses [19, 20, 26] indicated effect sizes of 0.5–0.6% reduction in HbA1c and 0.8–1 mmol/L reduction in FBG. Post hoc power calculations indicate that, with our sample size, we had over 95% power to detect effects of this magnitude. It is still possible that there is an effect of zinc but that it is markedly smaller in our Western population.

Three different meta-analyses all indicated a beneficial effect of zinc on glucose handling [18, 26, 27]. It is always possible that these differences are due to methodological deficits. In general, the studies included in these reviews are small, e.g. 12–40 people per arm, the heterogeneity of the pooled estimates is high, e.g. > 90%, and they did not check for publication bias, for example, using funnel plots or Egger’s test.

Although the studies included in these three reviews were different, there was a large body of overlap and a substantial number of these studies were conducted in non-Western countries, e.g. Iran, Iraq, India, Sri Lanka, where diets differ substantially. It is thus possible that zinc exerts a beneficial effect only in the setting of a zinc deficient diet. Given that zinc is found in the highest quantities in seafood (oysters and crab), meat (especially beef) and seeds/nuts (almonds, pepitas) [28] it is possible that an effect of zinc was seen in countries where vegetarian diets are more prominent, and that the effect of zinc was not seen in our Australian population due to a higher intake of meat [28].

Capdor et al. [27] pooled studies across normoglycaemic and dysglycemic populations, including metabolic syndrome, type 1 and type 2 diabetes, whereas Jayawardena and Wang limited the selection criteria more strictly to populations with diabetes. Another explanation is that diabetes leads to renal pathology and subsequently to loss of zinc through the kidneys; hence, zinc replacement in diabetic patients addresses a physiological deficiency and improves glucose handling. Previous studies have confirmed increased zinc loss in diabetes [29]; it appears that it is not necessary to have overt nephropathy for this to happen but that the presence of microalbuminuria is sufficient [30]. It is possible that the positive results from diabetes subjects skew the overall results in the Capdor meta-analysis? [27]. Nevertheless, the RCT of zinc in pre-diabetics by Ranasinghe et al. [20] is compelling in indicating that zinc does have a beneficial effect even before microalbuminuria is present, and likely points to the adequacy of the background diet as the key determinant of whether supplemental zinc influences glucose handling.

Another possibility is that the form of supplementation we used was inadequate. The majority of previous studies used zinc sulphate whereas we used zinc gluconate. Our choice was influenced by the greater tolerability of the gluconate salt and the greater bioavailability [31].

What then do we make of studies in Western populations that also show a beneficial effect? The majority of randomized trials included across the 3 meta-analyses from Western countries (e.g. Australia, France, USA) do not show favourable point estimates; it is only previous observational studies that show this. For example, our previous study looking at a community-based cohort of older Australians found that higher serum zinc (by 1 quartile) was associated with increased insulin sensitivity (by one decile) within the pre-diabetic group, even after adjusting for a wide range of potential confounders [32]. We also analysed data from the ‘Australian Longitudinal Study of Women’s Health’, a cohort of over 40,000 women across Australia, to determine whether dietary zinc was associated with risk of Type 2 Diabetes. In the stratum of middle-aged women, we found that those in the highest quintile of total dietary zinc intake had significantly lower risk of Type 2 Diabetes compared to the lowest quintile (OR = 0.50, 95% C.I. 0.32–0.77) [33]. In hindsight, perhaps the best interpretation of these results is that there was residual confounding, and that zinc (in the blood or diet) is a marker of a healthier diet and/or lifestyle that reduces the chance of developing diabetes but is not the key element on which to intervene in a Western population. This is further supported by the fact that an identical RCT protocol to the one in this study was tested in a Bangladeshi population and did show beneficial effects of zinc [34].

## Conclusions

Our study does carry some caveats. We did not reach our target sample size and the total number of participants recruited was modest relative to our recruitment target. Nevertheless, the methods were rigorous, and the randomisation was effective, leading to well-balanced groups. Almost 70% of participants gave complete data and adherence with tablets was over 85%, with zinc being very well tolerated. While a previous RCT indicated a positive effect of zinc in prediabetes [20], our results do not support this practice; the discrepancy may be related to whether the target population is zinc replete or zinc deficient.

## Supplementary Information

Below is the link to the electronic supplementary material.

Supplementary file1 (DOCX 13 kb)

## Data Availability

The datasets used and/or analysed during the current study are available from the corresponding author on reasonable request.

## Abbreviations

RCT: Randomised controlled trial
ZIPPeD: Zinc in preventing the progression of pre-diabetes
BMI: Body mass index
HbA1c: Haemoglobin A1c
FBG: Fasting blood glucose
HOMA 2: Homeostasis model assessment 2
REDCap: Research electronic data capture
CI, SD, IQR, OR: Confidence interval, standard deviation, Interquartile range, odds ratio
HNELHD: Hunter New England local health district
TRGS: Translational research grant scheme
HREC: Human research ethics committee
NSW: New South Wales
