# Supplementary material for: The effect of zinc supplementation on glucose homeostasis: a randomised double-blind placebo-controlled trial
Source: Acta Diabetol. 2022 Apr 22;59(7):965–75. doi: 10.1007/s00592-022-01888-x (PMC9026040; doi:10.1007/s00592-022-01888-x)
Supplement: Supplementary file 1 — Supplementary file1 (DOCX 13 kb) [file 592_2022_1888_MOESM1_ESM.docx]

***SupplemenataryTable***

***S1. Side effects***

|  | **Treatment group** | |
| --- | --- | --- |
|  | **Placebo (n=26)** | **Active (n=31)** |
| Dry mouth | 14 (54%) | 18 (58%) |
| Heartburn** | 11 (42%) | 13 (42%) |
| Indigestion | 11 (42%) | 7 (23%) |
| Stomach pain | 6 (23%) | 10 (32%) |
| Watery diarrhoea | 10 (38%) | 6 (19%) |
| Abdominal cramping | 5 (19%) | 10 (32%) |
| Taste in mouth | 7 (27%) | 7 (23%) |
| Vomiting | 1 (3.8%) | 2 (6.5%) |
| Other | 9 (35%) | 5 (16%) |
